# Artemisinin exerts anti-inflammatory effects in osteoarthritis through the inhibition of TGF-β1 signaling

**DOI:** 10.3389/fimmu.2026.1717045

**Published:** 2026-01-29

**Authors:** Xifan Zheng, Bo Yu, Yuansong Song, Yeping Chen, Zeming Li, Jun Yao

**Affiliations:** Bone and Joint Surgery, The First Affiliated Hospital of Guangxi Medical University, Nanning, Guangxi, China

**Keywords:** artemisinin, inflammatory factors, MMP13, osteoarthritis, TGF-β1

## Abstract

**Objective:**

Osteoarthritis (OA) involves an inflammatory imbalance, yet key mediators and their interplay with potential therapeutics like artemisinin (ART) remain poorly understood. This study aimed to systematically investigate these relationships using Mendelian randomization and to decipher their functional interactions through cellular and molecular experiments, complemented by network pharmacology and molecular docking analyses.

**Methods:**

Using the Mendelian randomization (MR) method, we integrated independent exposure–outcome genome-wide association study data to evaluate the causal association between inflammatory cytokines and OA. Chondrocytes were treated with IL-1β, TGF-β1 (5 μg/mL), and ART (4 μg/mL) for 24 hours. Cell proliferation was assessed using CCK-8 and EdU assays, and gene/protein expression was analyzed via RT-qPCR, Western blotting, and immunofluorescence staining. In parallel, network pharmacology was performed to identify putative ART targets related to OA and to characterize enriched pathways and hub genes through GO/KEGG enrichment and protein–protein interaction (PPI) analyses. Molecular docking was further conducted to evaluate the binding feasibility between ART and the catabolic mediator MMP-13.

**Results:**

MR revealed a positive association between TGF-β1 and OA risk (OR = 1.0526, P = 0.0182). Functionally, ART significantly enhanced chondrocyte proliferation, whereas TGF-β1 inhibited it. ART downregulated IL-1β and MMP13 expression, while TGF-β1 upregulated them, indicating opposing effects in OA chondrocytes. Network pharmacology suggested that ART-related OA targets were enriched in inflammation-associated processes and signaling pathways (e.g., MAPK signaling), with PPI analysis highlighting inflammatory signaling hubs (e.g., JAK/STAT-related nodes). Consistently, molecular docking demonstrated favorable binding of ART within the MMP-13 active pocket, supporting the structural feasibility of an ART–MMP-13 interaction.

**Conclusion:**

This study demonstrates that TGF-β1 plays an important pathogenic role in OA, as supported by MR and *in vitro* evidence, while ART exhibits anti-inflammatory and anti-catabolic effects by counteracting TGF-β1–driven inflammatory responses. Network pharmacology and docking analyses further suggest multi-target pathway regulation and a potential interaction with MMP-13. ART may represent a viable therapeutic candidate for OA; however, further studies are required to validate direct targets and elucidate tissue-specific mechanisms.

## 1 Introduction

OA is a progressive, chronic disorder marked by the degeneration of articular cartilage, synovial inflammation, and alterations in bone remodeling (1). Its pathological mechanism is closely related to the imbalance of inflammatory cytokines (2). Proinflammatory cytokines (e.g., IL-1β, TNF-α, and IL-6) are markedly elevated in the joint microenvironment of OA patients. These factors activate signaling pathways like NF-κB, prompting chondrocytes to produce matrix metalloproteinases (MMP-3, MMP-13) and aggrecanases (ADAMTS), thereby accelerating the degradation of the extracellular matrix (3–5). IL-1β or TNF stimulation can lead to glycolysis-related metabolic reprogramming in OA chondrocytes, characterized by mitochondrial respiration inhibition and an increased glycolysis rate. This process was verified by real-time metabolic analysis (Seahorse) and qPCR detection of metabolic genes (such as HK2 and LDHA) (6); Proinflammatory factors, including IL-1β, TNF-α, IL-6, and IL-17, enhance the synthesis of MMP-3/MMP-13 proteins by upregulating the expression of ERRγ (estrogen-related receptor γ). The ERRγ-specific inhibitor GSK5182 can effectively reverse this effect (4).

However, traditional observational studies have difficulty distinguishing the causal relationship between cytokines and OA. For example, synovial fluid testing has found that factors such as IL-1β and TNF-α are associated with the severity of OA. Still, it is impossible to determine whether they are the cause or consequence of OA (7). The Genetic Causal Inference Method uses genetic variation as an instrumental variable to avoid confounding bias and provide high-level evidence for causal inference: studies using this method have found that genetically predicted elevated levels of G-CSF (granulocyte colony-stimulating factor) can reduce the risk of knee OA, while elevated levels of MIP-1α (CCL3) are secondary to OA (8); macrophage colony-stimulating factor (MCSF) and vascular endothelial growth factor (VEGF) also have a causal relationship with the risk of knee OA (9).

Although previous studies have revealed the role of some inflammatory factors, there are still obvious deficiencies. First, most studies have focused on proinflammatory factors, while the role of anti-inflammatory factors such as TGF-β1 has not been systematically evaluated. TGF-β1 has a dual role in cartilage homeostasis, promoting cartilage repair and potentially aggravating fibrosis under certain conditions. Its specific mechanism in OA still needs to be further explored (10). Second, although natural compounds such as artemisinin have shown anti-inflammatory and antioxidant potential *in vitro* studies (for example, by activating the Nrf2/HO-1 pathway to inhibit NF-κB), their specific targets in OA and their interaction with TGF-β1 have not yet been clarified (10, 11). The Genetic Causal Inference Method also has obvious disadvantages. First, it relies on three core assumptions: genetic variation must be strongly associated with exposure (association hypothesis), unrelated to confounding factors (independence hypothesis), and only affect the outcome through exposure (exclusivity hypothesis). If these assumptions are not met, such as the existence of horizontal pleiotropy (i.e., genetic variation affects the outcome through other pathways), Genetic Causal Inference Method results may be biased (12, 13). Secondly, the Genetic Causal Inference Method can only provide causal evidence at the statistical level and cannot reveal specific biological mechanisms or pathological processes. Therefore, it must be verified through *in vitro* or *in vivo* experiments (12). For example, in OA research, the Genetic Causal Inference Method may identify a causal relationship between IL-1β and OA; however, cell experiments (such as chondrocyte culture) or animal models are needed to confirm its mechanism of promoting matrix degradation through the NF-κB pathway (10, 14). In addition, the effectiveness of the Genetic Causal Inference Method is limited by the strength of the genetic instrument variables and the sample size, which may lead to insufficient statistical power or false associations (13).

In summary, although previous studies have enriched our understanding of the relationship between inflammatory factors and OA, a research gap remains in the mechanism of action of TGF-β1 and artemisinin in OA, as well as their interrelationship. The Genetic Causal Inference Method is a powerful causal inference tool, but its results should be regarded as preliminary evidence and need to be combined with experimental studies (such as molecular biology experiments) to fully verify and clarify the mechanism (12, 13). Therefore, this study intends to explore the causal relationship between inflammatory cytokines and OA through Genetic Causal Inference Method analysis, and further verify the regulatory role of TGF-β1 and artemisinin in OA through molecular experiments, aiming to provide a new theoretical basis for targeted intervention of OA.

## 2 Methods

### 2.1 Data sources and study design

**Figure 1:**
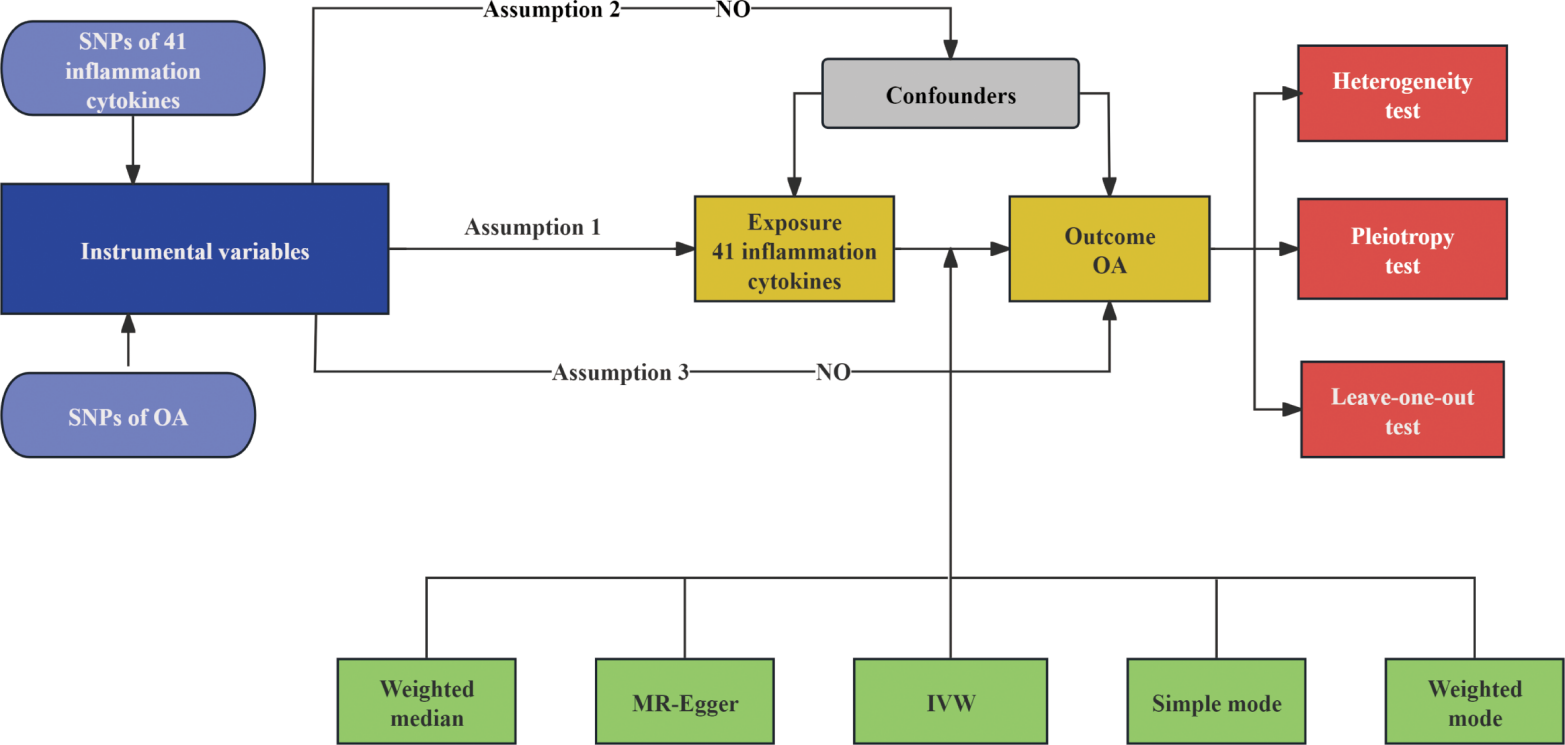
Workflow of the mendelian randomization study. The diagram illustrates the study design and analysis process, including the selection of genetic instruments for inflammatory cytokines, coordination with Osteoarthritis (OA) genome-wide association study (GWAS) data, primary inverse variance weighting (IVW) analysis, sensitivity analysis, evaluation of heterogeneity and pleiotropy, and robustness assessment through leave-one-out analysis.

This study used publicly available, de-identified GWAS summary statistics; therefore, no additional ethical approval or informed consent was required for the MR component, as these were obtained in the original studies. All animal procedures were approved by the Institutional Animal Care and Use Committee of the First Affiliated Hospital of Guangxi Medical University (Approval No. 2025-E0770) and were conducted in accordance with relevant guidelines. We performed a two-sample Mendelian randomization (MR) analysis as a genetic causal inference approach to assess the causal effect of the inflammatory cytokine TGF-β1 (GWAS ID: GCST90087933) (15), using independent exposure and outcome GWAS summary statistics. OA outcome data were obtained from the UK Biobank (GWAS Catalog ID: GCST90038686), comprising 39,515 cases and 445,083 controls of European ancestry. OA was defined based on hospital admission records (ICD-10 codes M15–M19) and self-reported diagnosis codes (16). Instrumental variants were required to be strongly associated with the exposure (relevance), independent of confounders (independence), and influence the outcome only through the exposure (exclusion restriction). The overall workflow is shown in **Figure 1**.

### 2.2 Selection of instrumental variables

Initially, a genome-wide significance threshold of P < 5 × 10^-8^ was set to identify single-nucleotide polymorphisms (SNPs) strongly associated with inflammatory cytokines and OA. However, due to the limited number of SNPs identified when considering inflammatory cytokines and OA as exposures, a slightly higher cutoff value of P < 5 × 10^-6^ was chosen (17). To ensure the selection of independent SNPs and minimize the impact of linkage disequilibrium (LD) on the results, an LD threshold of r² < 0.001 was applied, along with a genetic distance cutoff of 10,000 kb to guarantee independence across instrumental SNPs for each exposure (18). The strength of the association between the instrumental variables and the exposure was assessed using the F statistic. To minimize the bias introduced by weak instrumental variables, only SNPs with an F statistic greater than 10 were considered (19).

### 2.3 Extraction and culture of chondrocytes

An experimental approach was designed for preliminary validation of the trends from the MR analysis. In this study, chondrocytes were aseptically extracted and cultured from the knee cartilage tissue of 3- to 7-day-old Sprague-Dawley rats. Immediately after sacrifice, the knee joints were dissected, and the cartilage tissue was removed. Chondrocytes were released through a two-step enzymatic digestion using trypsin-EDTA and type II collagenase. Cells were collected by centrifugation and resuspended in high-glucose DMEM supplemented with 10% fetal bovine serum and 1% penicillin-streptomycin. The cells were seeded into T75 culture flasks and incubated at 37°C in a 5% CO_2_ atmosphere. After cell attachment, the medium was refreshed every 48 hours. When the cell density reached 80%-90%, cells were passaged using trypsin-EDTA, split at a 1:3 ratio, and cultured until the third passage for use in experiments. Strict aseptic procedures were adhered to throughout the extraction and culture process to ensure cell viability and experimental reliability.

### 2.4 Cell intervention experiments

To establish an *in vitro* OA model and assess the effects of TGF-β1 and artemisinin (ART) treatment, third-passage chondrocytes were stimulated with 10 ng/mL IL-1β (Beyotime, China). Cells were plated in 6-well plates at a density of 8 × 10^4^ cells/well and allowed to adhere for 24 hours before being grouped. The experimental groups included: a control group, an OA model group, an OA + ART (4 μg/mL, Aladdin, China) group, and an OA + TGF-β1 (5 μg/mL, Pepro Tech, USA) group. The intervention concentrations were selected based on prior CCK-8 assay results. The control group was maintained under standard culture conditions for 48 hours. The OA group was exposed to IL-1β for 24 hours, followed by 24 hours of routine culture. In the OA + ART and OA + TGF-β1 groups, after the initial 24-hour induction of IL-1β, the medium was replaced with fresh medium containing either 4 μg/mL ART or 5 μg/mL TGF-β1 for an additional 24 hours. To investigate whether ART could mitigate the pro-inflammatory or pro-fibrotic effects induced by TGF-β1, the treatment group was included. In the group, cells were first cultured with TGF-β1 for 24 hours after IL-1β induction, followed by a 24-hour incubation with ART.

### 2.5 Cell viability assay

Chondrocyte viability in response to TGF-β1 was assessed to determine an optimal treatment concentration. Third-passage chondrocytes were seeded in 96-well plates (1 × 10³ cells/well) and allowed to adhere for 24 h. Cells were then treated with serially diluted TGF-β1 (0.5–16 μg/mL) for 24 h. Subsequently, 10 μL of CCK-8 reagent (Beyotime, China) was added to each well and incubated for 2 h, and absorbance was measured at 450 nm. All experiments were performed in three independent biological replicates (n=3) with triplicate wells per condition.

### 2.6 EdU incorporation assay for cell proliferation analysis

Chondrocyte proliferation was evaluated using the BeyoClick™ EdU Cell Proliferation Assay Kit (Beyotime, China) according to the manufacturer’s instructions. In brief, cells were seeded at 3 × 10^5^ cells/well in 6-well plates and assigned to four treatment groups. Upon reaching 70–80% confluence, the cells were pulsed with 10 μM EdU for 2 hours. Subsequently, they were fixed with 4% paraformaldehyde (15 min), permeabilized with 0.3% Triton X-100 in PBS, and incubated with the Click reaction reagent for 1 hour at 37 °C. Nuclei were counterstained with Hoechst (Beyotime), and images were acquired using a Revolve2 inverted fluorescence microscope (Echo Laboratories, USA) to quantify EdU-positive cells. All experiments were performed in three independent biological replicates (n=3).

### 2.7 Total RNA extraction and RT-qPCR

qRT-PCR analysis was performed to evaluate the expression of inflammation-related genes in the four chondrocyte groups. Following RNA extraction (RNAeasy Animal RNA Isolation Kit, Beyotime, China) and reverse transcription (PrimeScript™ RT Master Mix, Takara, China), cDNA was amplified with PowerUp™ SYBR™ Green Master Mix on a 7500 Real-Time PCR System (both from Thermo Fisher Scientific) under the following conditions: 95°C for 30 sec; 40 cycles of 95°C for 10 sec and 60°C for 30 sec. Using GAPDH for normalization, relative expression levels were determined via the 2^(-ΔΔCt) method in triplicate assays. Primer sequences are listed in **Table 1**.

**Table 1:** Primer sequences.

Gene	Forward primer sequence	Reverse primer sequence
GAPDH	5'-CCATGGAGAAGGCTGGGG-3'	5'-TGGGTGGAATCATATTGGA-3'
MMP13	5'-GATGAGGCCAGGAGGAGTG-3'	5'-GAGAGGGTGAGGAGGTGAG-3'
IL-1β	5'-TGTCATCTCGGAGGATGAG-3'	5'-AGGGAAGTGGAGGTGAGGA-3'

This table presents the PCR primer sequences for GAPDH, MMP13, and IL-1β genes in rats, including both the forward (F) and reverse (R) sequences for each gene.

### 2.8 Western blotting

The protein expression levels of inflammatory mediators in chondrocytes were analyzed by Western blot. Briefly, cells from each group were lysed, and the supernatants were collected by centrifugation. Protein samples were denatured in SDS-PAGE loading buffer (Beyotime, China), and equal amounts of protein were resolved on 10% or 15% polyacrylamide gels (Epizyme Biotech, China) before being transferred to PVDF membranes (Epizyme Biotech, China). The membranes were blocked and subsequently incubated overnight with primary antibodies against IL-1β and MMP-13 (both from Proteintech, China), followed by incubation with an HRP-conjugated goat anti-rabbit secondary antibody (Proteintech, China). After washing with TBST (Solarbio, China), protein bands were visualized using an ultrasensitive ECL kit (Beyotime, China) on a Bio-Rad ChemiDoc imaging system. Band intensities were quantified with ImageJ software (NIH, USA). All experiments were performed in three independent biological replicates (n=3).

### 2.9 Immunofluorescence

To assess the localization and expression of inflammatory proteins, chondrocytes on coverslips were fixed, permeabilized, and blocked. The cells were then probed overnight at 4°C with specific primary antibodies (IL-1β or MMP-13; Proteintech, China). Subsequently, an FITC-labeled goat anti-rabbit IgG secondary antibody (Epizyme Biotech, China) was used for detection, and nuclei were visualized with DAPI. Fluorescence images were obtained using a Revolve2 microscope (Echo Laboratories, USA). For quantification, the mean fluorescence intensity (MFI) and the percentage of positive area were determined from threshold-selected regions in ImageJ (NIH, USA), and data were statistically compared among the experimental groups. Thresholds for positive staining were defined relative to the background fluorescence of negative controls to ensure specificity. The rate of positive cells in each section was measured by observers who were blinded to the experimental groups. All experiments were performed in three independent biological replicates (n=3).

### 2.10 Network pharmacology-based investigation of the mechanisms of artemisinin in the treatment of osteoarthritis

This study employed a network pharmacology approach to systematically investigate the potential mechanism of artemisinin in the treatment of osteoarthritis (OA). First, OA-related targets were retrieved from the GeneCards, OMIM, and Open Targets databases, while the potential targets of artemisinin were predicted using the ChEMBL, SuperPred, and SwissTargetPrediction databases. The intersection of the two sets was identified as the common targets. Subsequently, Gene Ontology (GO) annotation and Kyoto Encyclopedia of Genes and Genomes (KEGG) pathway enrichment analyses were performed to elucidate the associated biological processes and signaling pathways. Furthermore, a protein–protein interaction (PPI) network was constructed using the STRING database, and the top 30 key hub genes were screened based on degree centrality via the cytoHubba plugin in Cytoscape.

### 2.11 Molecular docking

Molecular docking simulations were conducted to evaluate the interactions between ART and proteins involved in inflammatory mediation. The three-dimensional structures of the target proteins were retrieved from the Protein Data Bank (PDB; https://www.rcsb.org/), while the chemical structure of ART was obtained from the PubChem database. Protein and ligand preparation was performed in a stepwise and standardized manner. Based on the active sites and known binding regions of the target proteins, the GetBox plugin was employed to define the binding pockets of the target proteins, thereby guiding the determination of the grid box center and dimensions. Subsequently, AutoDockTools was utilized to add polar hydrogen atoms, assign Gasteiger charges, and finalize the docking grid parameters. Molecular docking was then conducted using AutoDock Vina and AutoDock, with 100 independent docking runs performed for each protein–ligand pair. The docking pose exhibiting the lowest binding free energy was selected as the optimal conformation and saved in PDB format for further analysis. Finally, the Protein–Ligand Interaction Profiler (PLIP) web server and LigPlus software were employed to characterize the key interactions between the ligand and receptor and to generate high-quality visual representations of the docking results.

### 2.12 Investigation of the relationship between ART and TGF-β1 in osteoarthritis

The cells were divided into three groups and simultaneously stimulated with IL-1β to establish an OA chondrocyte model. The first group received ART, the second group received TGF-β1, and the third group received both ART and TGF-β1 stimulation. The expression of OA inflammatory markers was observed in the three groups using the aforementioned experimental procedures.

### 2.13 Statistical analysis

To estimate the causal effects of inflammatory cytokines on OA, we performed two-sample MR in R (v4.1.2) using the TwoSampleMR package. Random-effects IVW was the primary method, supported by MR-Egger, weighted median, and mode-based sensitivity analyses (20). Primary IVW P values were adjusted using the Benjamini–Hochberg FDR method (q < 0.05), with nominal P values also reported. Heterogeneity was assessed by Cochran’s Q (IVW) and Rucker’s Q (MR-Egger) (P > 0.05) (21), and horizontal pleiotropy by the MR-Egger intercept and MR-PRESSO global test (22). Leave-one-out analysis evaluated single-SNP influence (23). Data are shown as mean ± SD. For ≥3 groups, one-way ANOVA with Bonferroni correction was used (GraphPad Prism 9); two-group comparisons used unpaired two-tailed t-tests. P < 0.05 was considered significant unless stated otherwise. All *in vitro* experiments were conducted with ≥3 biological replicates (n=3), and RT-qPCR was run in technical triplicate.

## 3 Results

### 3.1 Genetic causal inference identified TGF-β1 linked to OA outcomes

**Figure 2:**
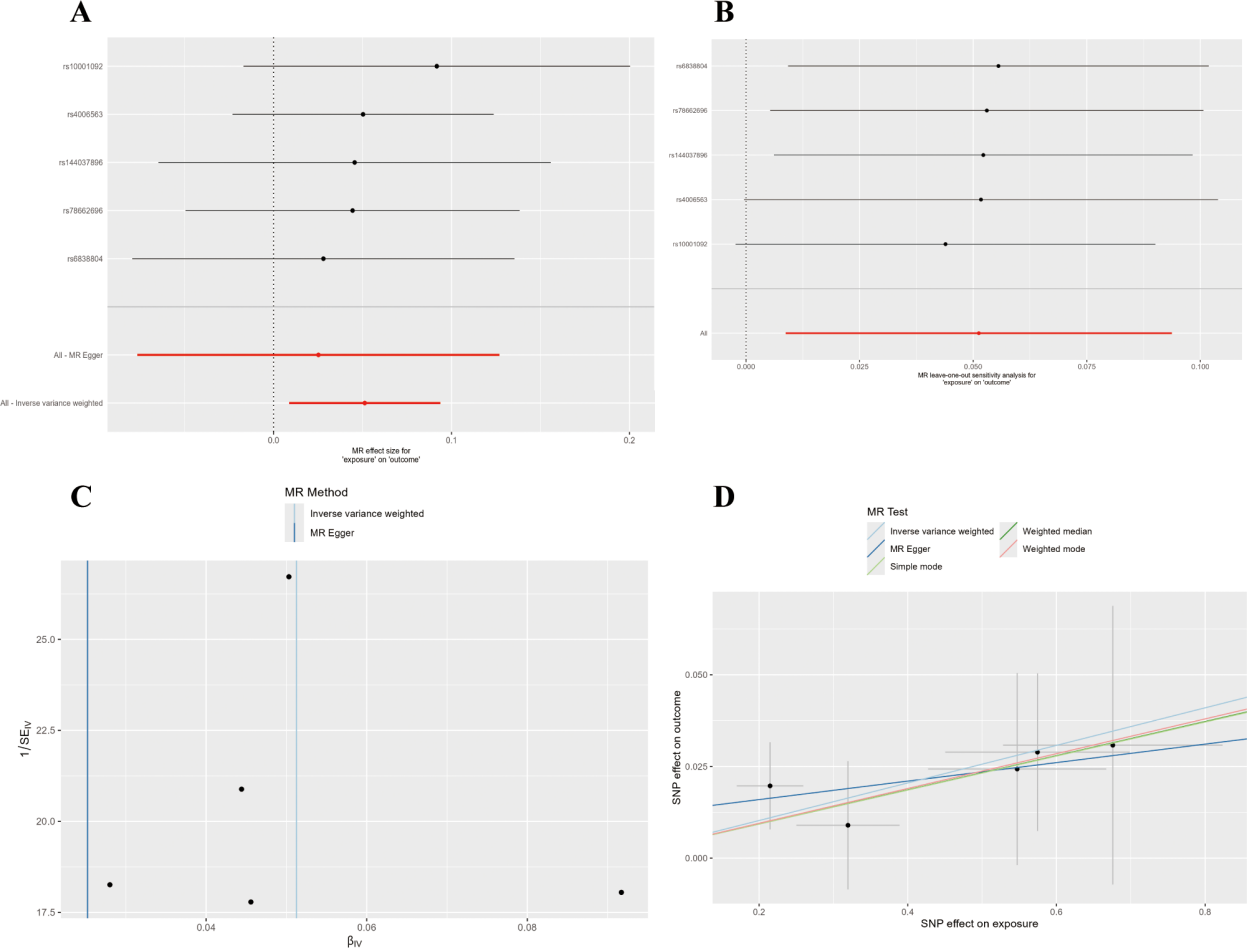
Mendelian randomization analysis of TGF-β1 on osteoarthritis: forest, sensitivity, funnel, and scatter plots. **(A)** Forest plot. **(B)** Leave-one-out sensitivity analysis plot. **(C)** Funnel plot **(D)** Scatter plot.

Osteoarthritis was investigated using a two-sample Mendelian randomization framework. Genetically predicted TGF-β1 showed a significant positive association with OA risk in the primary IVW analysis (OR = 1.0526, 95% CI: 1.0088–1.0983, P = 0.0182). Instrument information (including the number of SNPs, explained variance [R²], and mean/minimum F-statistics) is provided in **Supplementary Table 1** (TGF-β1: 9 SNPs; all F-statistics > 10). Sensitivity analyses supported robustness, with no evidence of heterogeneity (Cochran’s Q and Rucker’s Q, all P > 0.05) or horizontal pleiotropy (MR-Egger intercept and MR-PRESSO global test; **Supplementary Table 2**). Leave-one-out analysis further indicated that the association was not driven by any single SNP (**Figure 2**).

### 3.2 Optimal intervention concentrations of ART and TGF-β1 for OA chondrocytes

**Figure 3:**
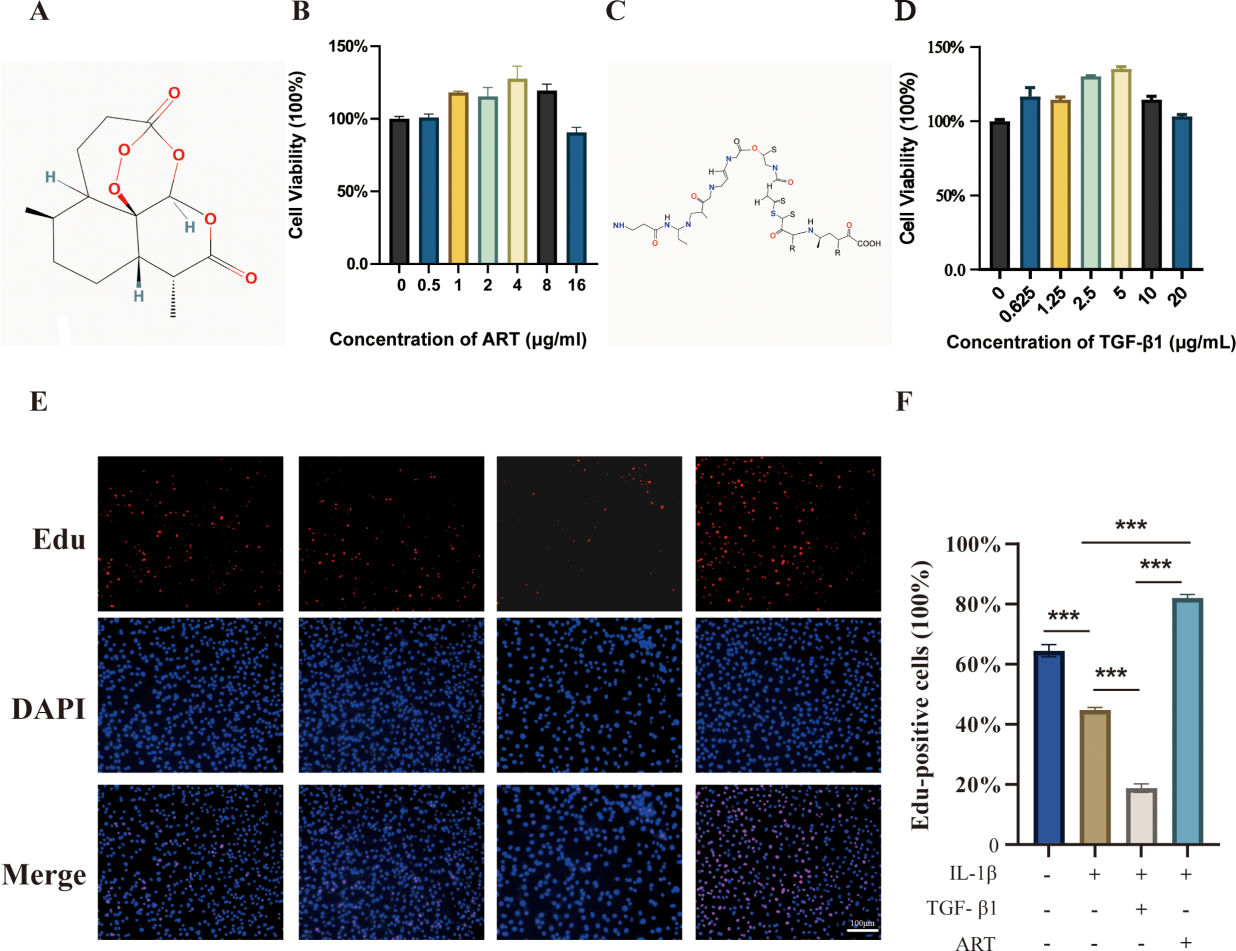
Effects of ART and TGF-β1 on chondrocyte viability and proliferation. **(A)** Chemical structure of Artemisinin (ART). **(B)** Alterations in chondrocyte viability following ART treatment at concentrations ranging from 0.5 to 16 μg/mL. **(C)** Chemical structure of Transforming Growth Factor-β1 (TGF-β1). **(D)** Changes in cell viability after treatment with TGF-β1 at concentrations ranging from 0 to 20 μg/mL. **(E)** EdU (red) and DAPI (blue) staining images illustrating the proliferative activity of chondrocytes in different treatment groups. Scalebar = 100 μm. **(F)** Quantification of the EdU-positive cell percentage in each treatment group. Data are presented as mean ± SD from three independent biological replicates (n=3). Statistical analysis was performed using one-way ANOVA followed by Bonferroni’s multiple-comparisons test. *P < 0.05, **P < 0.01, ***P < 0.001.

Cell proliferation was assessed using CCK-8 assays after treatment with different drug concentrations (**Figure 3**). In the ART-treated group (**Figure 3A**), low concentrations (0.5–4 μg/mL) did not significantly affect cell viability, while cell viability was significantly decreased in the 8 and 16 μg/mL treatment groups (**Figure 3B**). TGF-β1 treatment (**Figure 3C**) did not significantly inhibit cell viability within the 0.625–5 μg/mL range, but significantly decreased at 10 and 20 μg/mL (**Figure 3D**). ART at 4 μg/mL and TGF-β1 at 5 μg/mL were selected as the optimal concentrations for intervention. The results showed that ART enhanced chondrocyte viability, while TGF-β1 inhibited it.

### 3.3 ART and TGF-β1 have different effects on the proliferation of OA chondrocytes

The proportion of EdU-positive cells in the OA cell model group was significantly lower than that in the normal control group, indicating decreased cell proliferation (**Figures 3E, F**). TGF-β1 treatment further reduced the EdU-positive rate and inhibited cell proliferation (p < 0.01). ART treatment significantly increased the proportion of EdU-positive cells in the OA cell model, suggesting that ART can improve the proliferation of chondrocytes under OA conditions (p < 0.05).

### 3.4 Network pharmacology-based investigation of the mechanisms of artemisinin in the treatment of osteoarthritis

**Figure 4:**
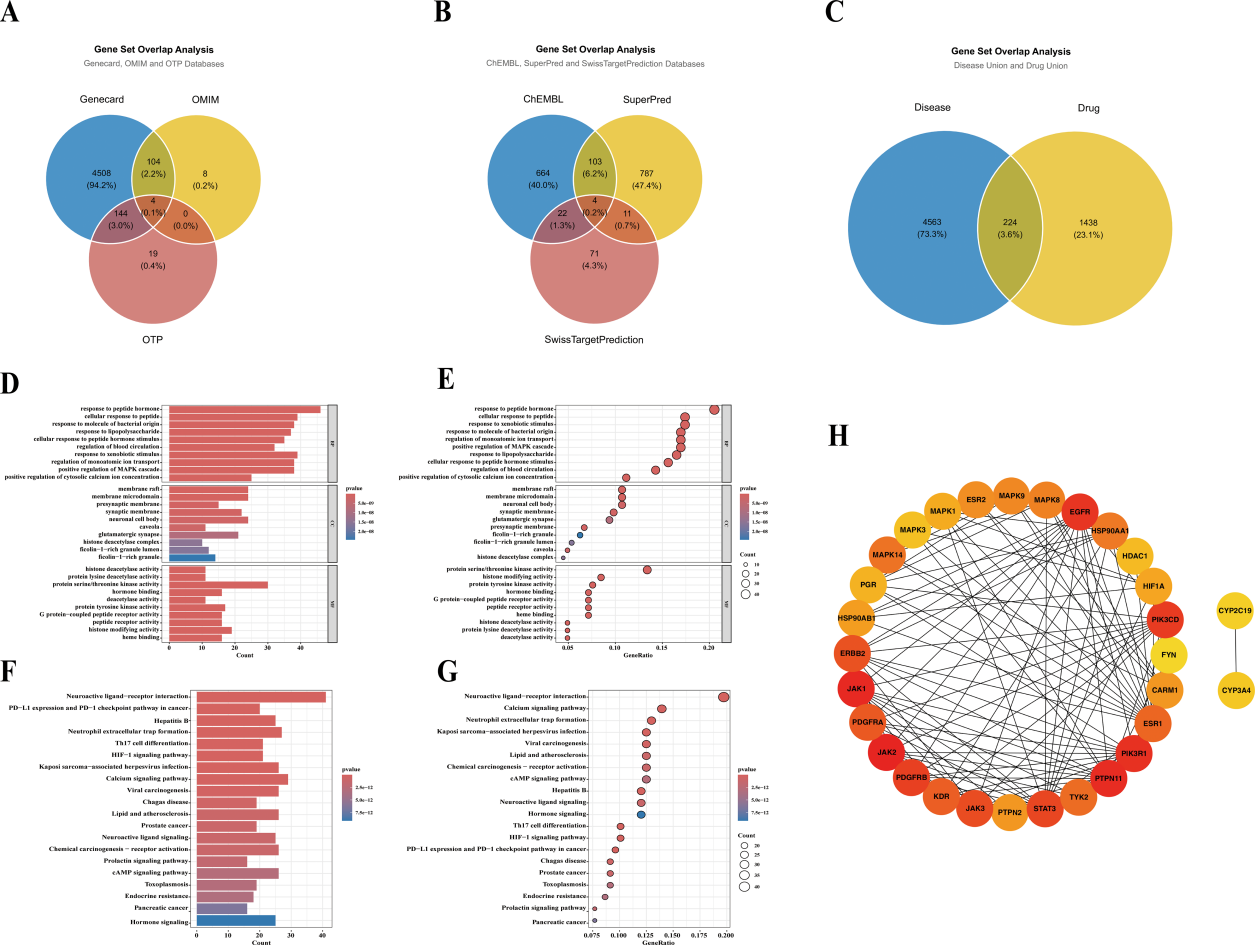
Network pharmacology-based analysis of potential targets of artemisinin against osteoarthritis. **(A)** Distribution of osteoarthritis-related targets from the GeneCards, OMIM, and Open Targets databases. **(B)** Distribution of artemisinin-related targets from the ChEMBL, SuperPred, and SwissTargetPrediction platforms. **(C)** Intersection of disease and drug targets, yielding the final common target set. **(D)** Bar plot of the top significantly enriched GO terms. **(E)** Dot plot of the enriched GO terms. **(F)** Bar plot of the top significantly enriched KEGG pathways. **(G)** Dot plot of the enriched KEGG pathways. **(H)** Top 30 core targets identified from the common targets using the Maximal Clique Centrality (MCC) algorithm. Color intensity represent MCC score.

Through multi-database searches, a total of 4,787 disease-related genes were obtained (GeneCards: 4,508; OMIM: 8; Open Targets: 19; common to all three: 4) (**Figure 4A**). Based on artemisinin, 1,662 drug targets were predicted (ChEMBL: 664; SuperPred: 787; SwissTargetPrediction: 71; common to all three: 4) (**Figure 4B**). The intersection of these two sets yielded 224 common targets, which will serve as the core focus for subsequent network pharmacology analysis (**Figure 4C**). GO (**Figures 4D, E**) and KEGG (**Figures 4F, G**) enrichment analyses revealed that these targets were significantly enriched in biological processes such as peptide hormone response and MAPK cascade regulation, as well as signaling pathways including neuroactive ligand-receptor interaction and the calcium signaling pathway. A PPI network was subsequently constructed, and the top 30 core targets were screened based on the Maximal Clique Centrality (MCC) algorithm (**Figure 4H**), among which JAK2, JAK1, PTPN11, PIK3R1, and EGFR ranked the highest (**Supplementary Table 3**).

### 3.5 Molecular docking

**Figure 5:**
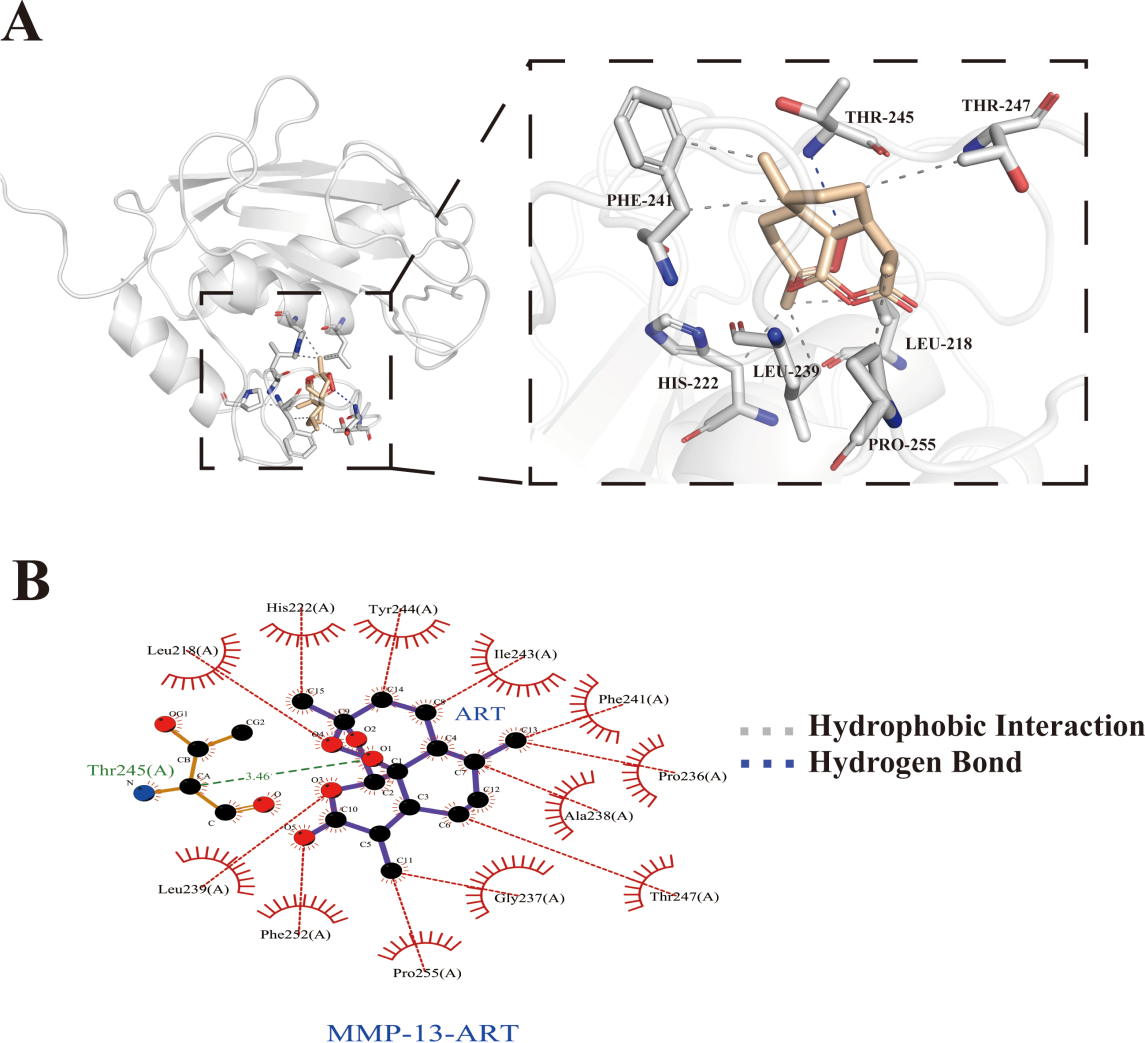
Molecular docking of ART with MMP-13. **(A)** Representation of the 3D interactions. **(B)** 2D interaction diagram.

Based on the active and binding sites of inflammation-related proteins, molecular docking analyses were conducted between ART and inflammatory mediator–associated proteins. The result showed that ART exhibited a favorable binding affinity toward MMP-13, with a binding energy of −8.94 kcal/mol (**Figure 5A**). Detailed interaction analysis revealed that ART formed hydrophobic interactions with the LEU-218, HIS-222, LEU-239, PHE-241, THR-247, and PRO-255 residues of MMP-13, along with a hydrogen bond involving the THR-245 residue. In addition, residues PRO-236, GLY-237, ALA-238, ILE-243, TYR-244, and PHE-245 of MMP-13 also participated in hydrophobic interactions with ART, as shown in **Figure 5B**.

### 3.6 TGF-β1 increases inflammatory gene expression, ART suppresses inflammatory gene expression

**Figure 6:**
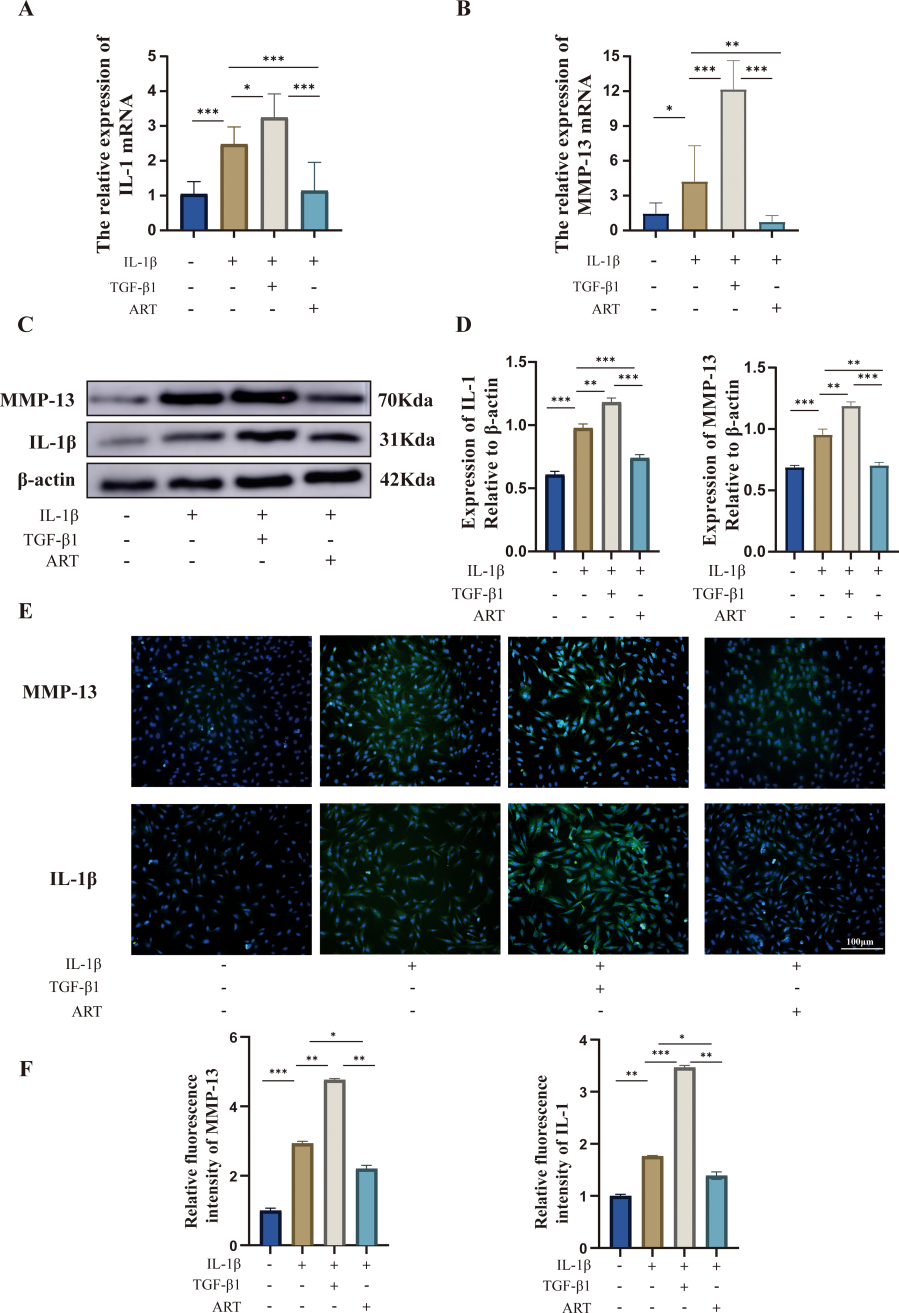
Effects of ART and TGF-β1 on the expression of IL-1β and MMP-13. **(A)** Relative mRNA expression of IL1B in each group, analyzed by RT-qPCR. **(B)** Relative mRNA expression of MMP13 in each group, analyzed by RT-qPCR. **(C)** Western blot analysis of IL-1β and MMP-13 protein expression. **(D)** Quantification of protein band intensity by densitometric analysis (normalized to β-actin). **(E)** Immunofluorescence staining showing the expression and localization of IL-1β and MMP-13 in cells (green indicates target protein, blue indicates DAPI nuclear staining). Scale bar = 100 μm. **(F)** Quantitative analysis of mean fluorescence intensity in immunofluorescence images. Data are presented as mean ± SD from three independent biological replicates (n=3). Statistical analysis was performed using one-way ANOVA followed by Bonferroni’s multiple-comparisons test. *P < 0.05, **P < 0.01, ***P < 0.001.

Compared with the control group, the expression of inflammatory markers IL-1β and MMP13 was significantly upregulated in OA-modeled chondrocytes (p < 0.001). In line with a pro-inflammatory role, TGF-β1 treatment further enhanced this expression, compared with the OA model group. Conversely, ART compared with the OA model group intervention effectively suppressed it (p < 0.01), suggesting a therapeutic potential for mitigating OA-related inflammation (**Figures 6A, B**).

### 3.7 Proinflammatory effects of TGF-β1 and anti-inflammatory effects of ART

At the protein level, TGF-β1 exhibited a pro-inflammatory role, which was effectively antagonized by ART. As evidenced by both Western blot (**Figures 6C, D**) and immunofluorescence (**Figures 6E, F**), the expression of IL-1β and MMP13 was significantly elevated in OA-modeled chondrocytes. TGF-β1 treatment amplified this increase, while ART treatment produced a significant reduction (p < 0.01), confirming their opposing functions in regulating inflammatory responses.

### 3.8 ART inhibits the inflammatory responses induced by TGF-β1 and exerts potent anti-inflammatory effects

**Figure 7:**
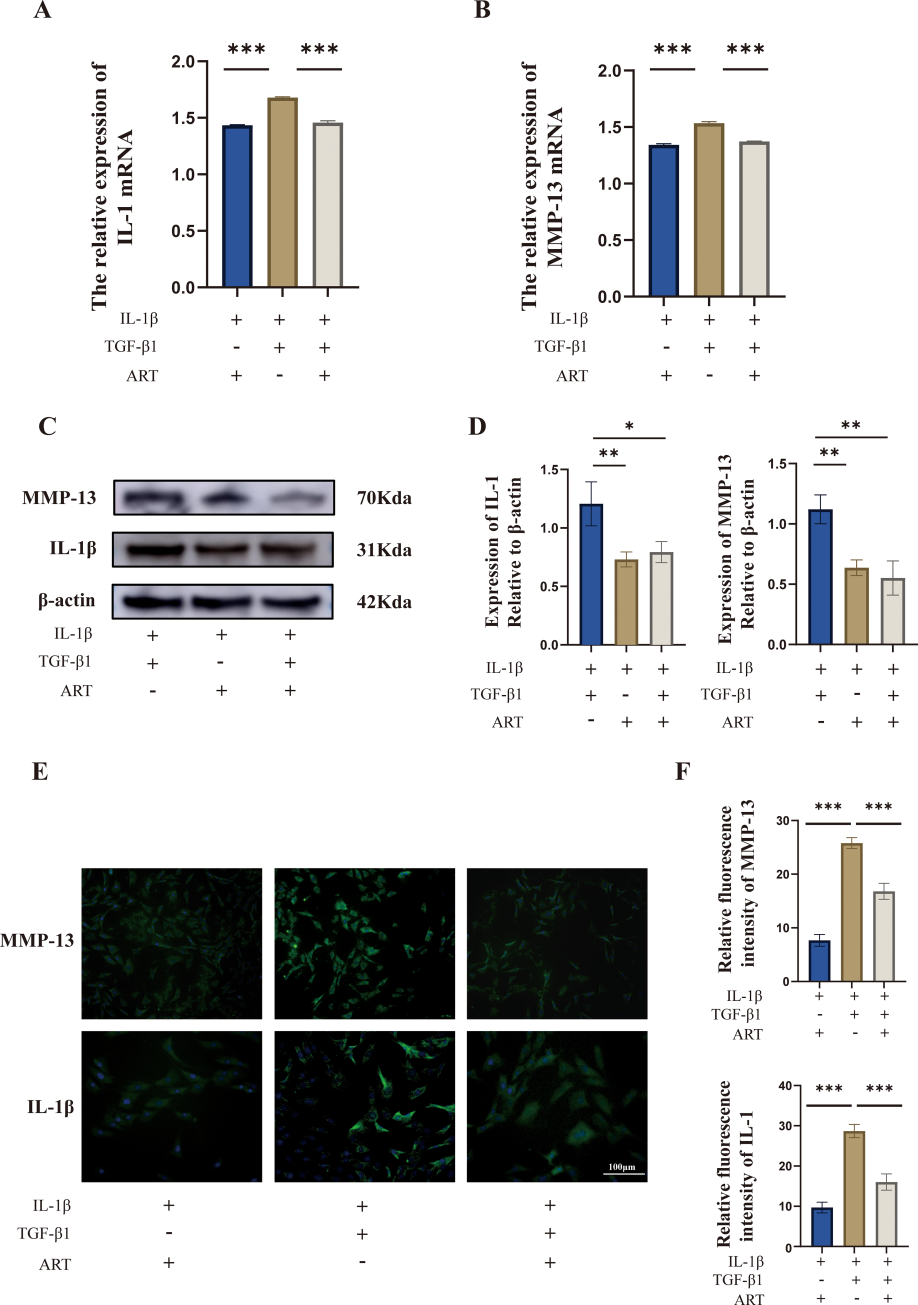
Artemisinin suppresses the pro-inflammatory effects of TGF-β1 in OA chondrocytes. **(A)** Relative mRNA expression of IL1B in each group, analyzed by RT-qPCR. **(B)** Relative mRNA expression of MMP13 in each group, analyzed by RT-qPCR. **(C)** Western blot analysis of IL-1β and MMP-13 protein expression. **(D)** Quantification of protein band intensity by densitometric analysis (normalized to β-actin). **(E)** Immunofluorescence staining showing the expression and localization of IL-1β and MMP-13 (green indicates target protein; blue indicates DAPI nuclear staining). Scale bar = 100 μm. **(F)** Quantitative analysis of mean fluorescence intensity in immunofluorescence images. Data are presented as mean ± SD from three independent biological replicates (n=3). Statistical analysis was performed using one-way ANOVA followed by Bonferroni’s multiple-comparisons test. *P < 0.05, **P < 0.01, ***P < 0.001.

Using PCR (**Figures 7A, B**), WB (**Figures 7C, D**), and immunofluorescence (**Figures 7E, F**) assays, we observed that IL-1β induced an inflammatory response in chondrocytes, which TGF-β1 further exacerbated. ART intervention alleviated the inflammatory response triggered by IL-1β and also antagonized the pro-inflammatory effects of TGF-β1; however, the mechanism of this antagonism remains unclear, whether it is direct or indirect. These results suggest that ART exerts a mitigating effect on both cytokine-driven inflammation and TGF-β1-related inflammatory pathways (p < 0.05).

## 4 Discussion

We used a genetic causal inference framework (two-sample Mendelian randomization, MR) to assess the causal relationship between TGF-β1 and OA risk. Using IVW as the primary estimator, we observed a significant positive association between genetically predicted TGF-β1 and OA risk (OR = 1.0526, P = 0.0182). This direction is broadly in line with certain prior population-level observations, although the finding warrants cautious interpretation. Notably, the TGF-β1 exposure captured by genetic instruments may represent a composite of different isoforms and/or activation states (24). In this context, the MR signal is more reflective of circulating “total TGF-β1” (i.e., integrated exposure encompassing both latent and active forms), whereas many cellular and tissue-based studies—including our *in vitro* experiments—primarily interrogate the effects of locally activated TGF-β1 within the joint microenvironment. This “exposure phenotype discrepancy” is biologically consequential: latency-associated peptide TGF-β1 (TGF-β1 LAP) may exert protective effects in OA, whereas full-length/aberrantly activated TGF-β1 under pathological conditions may exacerbate joint damage through pro-fibrotic processes (25, 26). Accordingly, the apparent discordance between genetic associations and certain experimental phenotypes is more plausibly explained by the non-equivalence between circulating exposure proxies and the locally activated state/dose gradient within the joint, rather than mutually exclusive conclusions. Consistent with this interpretation, TGF-β1 expression can be markedly elevated in OA tissues, and bone marrow mesenchymal stem cell (BMMSC) interventions have been reported to downregulate TGF-β1 and alleviate joint inflammation (27). Importantly, such observations may reflect a resetting of the inflammation–repair equilibrium in specific models or disease stages, rather than implying a strictly unidirectional pathogenic role of TGF-β1 in OA. Collectively, these considerations suggest that genetically predicted elevations in TGF-β1 may also relate to compensatory remodeling/repair responses during degeneration, with the direction and magnitude of its biological effects potentially varying across disease stages and severity—an issue that merits further clarification.

Against this background, accumulating evidence indicates that restoring anti-inflammatory homeostasis may confer protective benefits in OA. In line with this concept, our *in vitro* data show that artemisinin (ART) attenuates inflammatory and catabolic responses in OA chondrocytes, as evidenced by downregulation of key mediators such as IL-1β and MMP13. Prior studies likewise highlight the therapeutic potential of targeting pivotal inflammatory axes (28–30): BMMSC treatment in animal OA models has been reported to reduce serum pro-inflammatory cytokines (TNF-α and IL-17) while increasing the anti-inflammatory cytokine IL-4; moreover, TG-C tissue gene therapy incorporating TGF-β1–modified cells can promote M2 macrophage polarization, characterized by upregulation of IL-10 and IL-1ra and suppression of TNF-α, thereby improving the inflammatory milieu (29). Although ART may exert anti-inflammatory activity through multiple pathways (e.g., mechanisms related to PGE2) and may not be strictly dependent on TGF-β1 (29), our genetic causal inference prioritizes TGF-β1 as a key mediator significantly associated with OA risk, providing human genetic support for subsequent mechanistic validation centered on a TGF-β1–related inflammatory axis.

Our selection of ART is further supported by translational considerations. As a natural product with a long history of clinical use, ART offers practical advantages including a relatively established safety foundation and broad accessibility (31). In addition, ART and its derivatives have demonstrated immunomodulatory potential across diverse inflammatory models, influencing cytokine expression, oxidative stress status, and immune cell function—features that align with the prevailing view of OA as a degenerative disease driven by low-grade chronic inflammation (32). To obtain a systems-level perspective on ART’s potential mechanisms, we further performed network pharmacology analyses. ART-predicted targets were significantly enriched in inflammation-related biological processes and the MAPK signaling cascade (33), and were also enriched in calcium signaling and neuroactive ligand–receptor interaction pathways that are closely linked to cartilage homeostasis and mechanotransduction (34). PPI network analysis further identified JAK1 and JAK2 as hub targets, implicating a potential involvement of the JAK/STAT axis, which is recognized as an important mediator of inflammatory cytokine signaling in OA and an emerging therapeutic target (35). Consistently, inhibition of JAK signaling has been reported to alleviate synovial inflammation and exert chondroprotective effects in OA models (36). Together, these system-level lines of evidence suggest that ART may act via multi-target regulation of inflammatory, immune, and mechanotransduction-related pathways, and provide a broader mechanistic context for our subsequent focus on the MR-prioritized TGF-β1–related inflammatory axis.

Functionally, we observed that higher-dose TGF-β1 markedly suppressed chondrocyte proliferation and was accompanied by upregulation of catabolism-associated markers, supporting the notion that pathologically activated TGF-β1 may disrupt cartilage homeostasis; this is consistent with prior *in vivo* observations in which elevated TGF-β1 expression aggravated cartilage degeneration (30). In contrast, low-dose ART exhibited no appreciable cytotoxicity and significantly increased the fraction of EdU-positive cells, indicating enhanced proliferative potential. Importantly, under TGF-β1 stimulation, ART still substantially blunted TGF-β1–induced inflammatory and catabolic responses: it reduced IL-1β mRNA expression and decreased MMP13 protein levels, accompanied by concordant attenuation of immunofluorescence signals, supporting a clear antagonistic and chondroprotective effect in a TGF-β1–driven inflammatory–catabolic context. Furthermore, molecular docking was used to evaluate a potential ART–MMP-13 interaction. ART adopted a stable binding pose within the MMP-13 active pocket, and several key residues overlapped with those reported for selective MMP-13 inhibitors (37, 38). This provides a structural feasibility clue that ART may interact with MMP-13, suggesting that MMP-13 could represent a candidate binding protein/candidate target associated with ART’s anti-catabolic effects; however, this structural inference requires further validation using binding assays and functional perturbation approaches (e.g., pathway blockade or genetic interventions). Overall, these findings indicate that ART can antagonize the TGF-β1–driven “inflammation–catabolism” program and confer chondroprotective effects, while the direct molecular targets and critical pathway nodes remain to be elucidated.

Mechanistically, prior studies suggest that ART may modulate TGF-β biology through multiple routes, including reducing TGF-β release, inhibiting TGF-β signaling transmission, attenuating pathological angiogenesis, and potentially interacting with TGF-β receptors (39). These reported mechanisms, together with our MR prioritization of TGF-β1 and *in vitro* validation, support the translational relevance of an ART–TGF-β axis in OA intervention. Nevertheless, several limitations warrant consideration. Genetic causal inference primarily relies on genetic proxies for circulating inflammatory proteins, whereas the core pathology of OA resides within the local joint compartment; synovial fluid TGF-β1 concentrations may substantially exceed those in peripheral circulation, and locally activated forms may more directly drive cartilage degradation (30). Moreover, MR captures static genetic effects and cannot fully reflect dynamic changes during OA progression, such as the conversion of TGF-β1 from latent to active states (28, 29). Although sensitivity analyses did not indicate substantial heterogeneity or horizontal pleiotropy, residual bias cannot be completely excluded; additionally, genetic instruments may influence OA risk through non-inflammatory pathways (e.g., metabolic or mechanical stress–related routes), which MR cannot definitively rule out (28).

## 5 Conclusions

By integrating genetic causal inference (two-sample MR) with experimental evidence, our study provides convergent support for a potential pathogenic role of TGF-β1 in OA progression at both the human genetic and *in vitro* functional levels. In parallel, our *in vitro* data indicate that ART exerts anti-inflammatory and anti-catabolic effects in chondrocytes and can partially attenuate TGF-β1–induced pro-inflammatory and catabolic responses. Although the direct molecular targets and pathway-level mechanisms underlying these interactions remain to be fully defined, the genetic and experimental consistency implicating the TGF-β1–associated inflammatory axis highlights a promising avenue for targeted intervention in OA. Future studies should validate the molecular forms and local activation status of TGF-β1 in human joint tissues (e.g., cartilage and synovium) and delineate the spatiotemporal and tissue-specific mechanisms by which ART modulates this axis.

## Data Availability

The datasets presented in this study can be found in online repositories. The names of the repository/repositories and accession number(s) can be found in the article/**Supplementary Material**.
