# Supplementary material for: Artemisinin exerts anti-inflammatory effects in osteoarthritis through the inhibition of TGF-β1 signaling
Source: Front Immunol. 2026 Jan 29;17:1717045. doi: 10.3389/fimmu.2026.1717045 (PMC12895686; doi:10.3389/fimmu.2026.1717045)
Supplement: Supplementary file 1 [file Supplementaryfile1.docx]

**Index**

**Supplementary Table S1.** Summary statistics of the instrumental variables associated with TGF-β1 and their effects on osteoarthritis.................................................................................................................................................................................................................2

**Supplementary Table S2.** Summary of heterogeneity and pleiotropy test results for TGF-β1 ......................................................................................................................................................................................................................................3

**Supplementary Table S3.** Top 30 genes ranked by MCC method ...................................................................................................................................................................................................................................4-5

**Supplementary Table S1.** Summary statistics of the instrumental variables associated with TGF-β1 and their effects on osteoarthritis

| **exposure** | **outcome** | **id.exposure** | **id.outcome** | **SNP** | **b** | **se** | **p** | **or** | **or_lci95** | **or_uci95** | **R^2^** | **F** |
| --- | --- | --- | --- | --- | --- | --- | --- | --- | --- | --- | --- | --- |
| TGF-β1 | Osteoarthritis | GCST90087933 | GCST90038699 | rs11153642 | 0.002970572 | 0.001983774 | 0.134280136 | 1.002974988 | 0.999082796 | 1.006882343 | 0.023118378 | 19.47669499 |
| TGF-β1 | Osteoarthritis | GCST90087933 | GCST90038699 | rs142011798 | 0.001727542 | 0.00189084 | 0.360907335 | 1.001729035 | 0.998023452 | 1.005448377 | 0.000755816 | 19.51632763 |
| TGF-β1 | Osteoarthritis | GCST90087933 | GCST90038699 | rs147229052 | 0.002523366 | 0.003153599 | 0.423621336 | 1.002526553 | 0.996348993 | 1.008742414 | 0.001559599 | 35.88775874 |
| TGF-β1 | Osteoarthritis | GCST90087933 | GCST90038699 | rs17097452 | -7.60E-04 | 0.002349432 | 0.746475163 | 0.999240739 | 0.994649926 | 1.003852741 | 0.001155837 | 35.88976665 |
| TGF-β1 | Osteoarthritis | GCST90087933 | GCST90038699 | rs17211115 | 0.003616937 | 0.002284839 | 0.113417849 | 1.003623486 | 0.999139023 | 1.008128077 | 0.001407616 | 35.89405476 |
| TGF-β1 | Osteoarthritis | GCST90087933 | GCST90038699 | rs2485176 | 5.29E-04 | 0.002186603 | 0.808891066 | 1.000528985 | 0.996250152 | 1.004826194 | 0.000728175 | 23.00303628 |
| TGF-β1 | Osteoarthritis | GCST90087933 | GCST90038699 | rs3814360 | -4.70E-04 | 0.001962872 | 0.810886301 | 0.999530429 | 0.995692394 | 1.003383258 | 0.000789471 | 22.97989702 |
| TGF-β1 | Osteoarthritis | GCST90087933 | GCST90038699 | rs6089984 | 0.003901118 | 0.002107195 | 0.064121784 | 1.003908738 | 0.999771042 | 1.008063558 | 0.000952355 | 20.01566428 |
| TGF-β1 | Osteoarthritis | GCST90087933 | GCST90038699 | rs9398519 | 3.20E-04 | 0.001812653 | 0.860030149 | 1.000319687 | 0.996772057 | 1.003879942 | 0.000952355 | 20.01566428 |

**Note:** SNP, single nucleotide polymorphism; OR, odds ratio; CI, confidence interval; R^2,^ variance explained by the SNP; F, F-statistic for instrument strength.

**Supplementary Table S2.** Summary of heterogeneity and pleiotropy test results for TGF-β1

| **Exposure** | **Outcome** | **Heterogeneity's test** | **Pleiotropy's test** |
| --- | --- | --- | --- |
| **TGF-β1** | OA | 0.946 | 0.620 |
|  |  |  |  |
|  |  |  |  |
|  |  |  |  |
|  |  |  |  |

**Supplementary Table S3.** Top 30 genes ranked by MCC method

| **Rank** | **Name** | **Score** |
| --- | --- | --- |
| 1 | JAK2 | 21133 |
| 2 | JAK1 | 21120 |
| 3 | PTPN11 | 21041 |
| 4 | PIK3R1 | 20963 |
| 5 | EGFR | 20300 |
| 6 | PIK3CD | 10848 |
| 7 | PDGFRB | 10800 |
| 8 | STAT3 | 10465 |
| 9 | JAK3 | 10321 |
| 10 | ERBB2 | 10188 |
| 11 | PDGFRA | 10080 |
| 12 | KDR | 729 |
| 13 | ESR1 | 411 |
| 14 | TYK2 | 240 |
| 15 | MAPK14 | 199 |
| 16 | HSP90AA1 | 193 |
| 17 | MAPK8 | 177 |
| 18 | MAPK9 | 170 |
| 19 | ESR2 | 147 |
| 20 | PTPN2 | 120 |
| 20 | CARM1 | 120 |
| 22 | HSP90AB1 | 108 |
| 23 | HIF1A | 95 |
| 24 | MAPK1 | 78 |
| 25 | PGR | 60 |
| 26 | HDAC1 | 53 |
| 27 | MAPK3 | 52 |
| 28 | CYP3A4 | 49 |
| 29 | CYP2C19 | 36 |
| 30 | FYN | 34 |
